# Evaluation of Antimicrobial or Non-antimicrobial Treatments in Commercial Feedlot Cattle With Mild Bovine Respiratory Disease Based on a Refined Case-Definition

**DOI:** 10.3389/fvets.2020.571697

**Published:** 2020-10-07

**Authors:** Jason Nickell, Lonty Bryant, Kelly F. Lechtenberg, Charley Cull

**Affiliations:** ^1^Allflex Livestock Intelligence, Madison, WI, United States; ^2^Merck Animal Health, De Soto, KS, United States; ^3^Midwest Veterinary Services, Oakland, NE, United States; ^4^Veterinary and Biomedical Research Center, Manhattan, KS, United States

**Keywords:** bovine respiratory disease complex, tildipirosin, flunixin transdermal solution, cattle, diagnosis, negative control

## Abstract

The study objective was to compare clinical and performance outcomes among feedlot steers treated for bovine respiratory disease (BRD) with tildipirosin (TIL), flunixin transdermal solution (FTS; topical application), or both, based on a refined BRD case-definition. Crossbred steer calves (*N* = 2,380) were enrolled based on a clinical illness score (CIS) of 1–3; a rectal temperature between >102.5° F and ≤103.9° F; and a Whisper Score (WS) = 1 or ≥2. Within each WS stratum, steers were randomly allocated to Saline, TIL, FTS, or TIL + FTS to reflect a 2 × 2 factorial design. Individual health and performance outcomes were measured on Day 60 and closeout. From Day 0 through Day 60, in both strata, TIL resulted in significantly (*P* ≤ 0.05) fewer BRD retreatment events, fewer 3rd BRD treatments, fewer steers that did not finish, and greater average daily gain when compared to steers that were not treated with TIL. From Day 0 through closeout, cattle with a WS ≥ 2, treated with TIL had fewer animals (*P* ≤ 0.05) that did not finish compared to steers not treated with TIL. In this study, feedlot steers with clinical signs of BRD and rectal temperatures lower than traditional cutoffs displayed a positive response to antimicrobial therapy. A clear benefit of FTS was not observed in this study. Calves with a WS ≥ 2 were lighter at the time of first BRD treatment compared to calves with a WS = 1. However, standalone TIL therapy was the optimal BRD treatment modality across WS strata in this study.

## Introduction

The traditional case-definition for bovine respiratory disease (BRD) diagnosis in feedlot cattle reflects an animal exhibiting clinical signs of BRD such as anorexia, depression, nasal discharge, cough, respiratory difficulty; and, rectal temperature >104° F (1–6). This temperature threshold is also utilized in the BRD case-definition within the regulatory approval process for antibiotics in the United States (7–11). However, the diagnostic accuracy of that case-definition has previously been shown to be relatively poor (12, 13).

Antimicrobial therapy is indicated for an animal meeting those above criteria for a BRD diagnosis. Tildipirosin (TIL) is an antimicrobial medication indicated for treatment and control of bovine respiratory disease (BRD) associated with *M. haemolytica, P. multocida*, and *H. somni*. This drug has previously shown efficacy when applied to animals meeting the above BRD case-definition (14).

In practice, an animal that exhibits a sufficient clinical illness score (CIS) but fails to meet this specific threshold criterium for rectal temperature may be returned to its home-pen without treatment. Cattle that do not fit the case-definition, by not meeting the threshold for rectal temperature, may be misclassified or mistreated; thereby, jeopardizing animal well-being (not treating an animal that really is sick), misusing medication (wasting medication by treating an animal that won't benefit), subsequently impacting animal performance and profitability. Little data are available regarding “response to treatment” of cattle that have those clinical signs and rectal temperature ≤ 103.9° F. An additional question that has yet to be answered is if cattle that exhibit a sufficient clinical illness score but fail to meet the rectal temperature criteria of ≥104° F have the potential to respond to non-antimicrobial therapy. Flunixin meglumine Transdermal Solution (FTS) is indicated for control of pyrexia associated with bovine respiratory disease and the control of pain associated with foot rot. Using the conventional BRD case definition, utilizing FTS as an ancillary therapy (i.e., in addition to antimicrobial therapy) has not provided added value compared to the antimicrobial alone (15). However, to date, no data are available (to the authors' knowledge) that has assessed FTS as a standalone therapy among animals that exhibit clinical signs of BRD but fail to meet a traditional rectal temperature cutoff.

Given the perceived lack of accuracy afforded by current BRD diagnostic modalities, additional information (i.e., in addition to CIS and rectal temperature) may be necessary to improve overall accuracy while potentially improving the treatment decision-making process. Whisper® technology offers unique information that estimates the lung health of an individual calf at the time it has been identified with a tentative BRD diagnosis (16–19).

The objective of this study was to use a refined case-definition of BRD based on CIS, rectal temperature (≥102.5° F to ≤ 103.9° F) and results of computer-assisted lung auscultation (Whisper®) to compare clinical and performance outcomes of cattle treated because of BRD. The null hypothesis was that calves meeting the refined BRD case definition would not respond to antimicrobial, non-antimicrobial, or a combination of both therapies.

## Materials and Methods

The study protocol was submitted to the MVS Institution for Animal Care and Use Committee (IACUC) where the protocol received approval on 30 January 2018. The assigned IACUC number is AC17100B.

### Animals

The same study protocol was followed at each of two study sites (Oakland, NE; Manhattan, KS). Beef or beef-cross feeder steers (*N* = 3,376) with moderate to high risk for developing BRD were procured through several livestock auctions in Nebraska, Iowa, South Dakota, and Missouri across 24 shipments from late February to late November of 2018. All steers were weaned at the time of procurement; and, the history of vaccination or treatments was not known. When the steers arrived at the study site, their health was evaluated; they were identified with an individual number; they were vaccinated (modified-live viral, multi-valent clostridial toxoid), dewormed, and implanted with a growth-promotant; and, weighed. No antimicrobials were administered to control BRD (metaphylaxis). Steers that were not healthy were not eligible for enrollment which occurred from March through December of 2018. Steers were housed in open-air, dirt-floor pens. Conditions and management of the pens were according to standard feedlot practices. All steers were fed a ration appropriate for the size, age, and stage of feeding. After a brief step up period (~1 week), cattle were fed once daily a finisher diet, which included (DM basis): 54.1% high moisture corn, 25.3% wet distillers grain, 12.6% sweat brand 60, 3.4% corn stalks, 2.6% liquid supplement, and 2.0 micromineral mix ingredients [including 400 mg of monensin/animal and 85 mg of tylosin/animal per day (Elanco Animal Health, Greenfield, IN)]. Fresh feed was delivered each morning. Fresh water was available *ad libitum*. Waterers were monitored daily and cleaned when necessary. Any health-related intervention not described in the study protocol was administered at the discretion of the attending veterinarian after consultation with the sponsor. The monitor of the study was notified when such treatments were administered or when any steer was euthanized. The attending veterinarian at each site performed a necropsy on any steer found dead or was euthanized. If BRD was diagnosed at necropsy during the first 60 days of the study (Day 0 to Day 60), real-time polymerase chain reaction (RT-PCR) was performed with samples of lung to identify the following specific pathogens: infectious bovine rhinotracheitis (IBR) virus, bovine viral diarrhea virus (BVDV), bovine parainfluenza virus type 3 (PI3), bovine respiratory syncytial virus (BRSV), bovine influenza virus (BIV), *Mannheimia haemolytica, Pasteurella multocida, Histophilus somni*, and *Mycoplasma bovis*.

### Inclusion Criteria

Twenty-four hours after processing at arrival, steers were eligible for enrollment. The case-definition of BRD, refined for this study, was a clinical illness score (CIS) of 1, 2, or 3; rectal temperature >102.5° F and ≤ 103.9° F; and, a Whisper® score (WS) of 1 or ≥2. The CIS was assigned a number (0–4) based on the description in Table 1. Rectal temperatures were measured with digital GLA Agricultural Electronics thermometers that were calibrated prior to the study. The Whisper® Veterinary Stethoscope is a computerized stethoscope that measures and analyzes the sounds of the lungs and heart of individual animals using a machine-learning algorithm that assigns a score of 1 through 5 and estimates lung health at the time of clinical disease identification with increasing severity as scores rise (16–19). A lung health estimate of 1 indicates that the lung tissue of the respective calf is relatively healthy. Conversely, a lung health estimate of 5 reflects severely compromised lung tissue. Scores 2–4 reflect intermediary changes in lung health as the scale increases. The bell of the stethoscope was placed approximately two inches caudal and dorsal to the point of the right elbow of the calf. The area was cleaned if needed and sounds were recorded for 8 s. If the recording was not acceptable (“flagged” by the computer; < M60% of the entire recording present on the computer screen; or, the operator had reason to consider the recording to be inadequate), another recording was obtained.

**Table 1:** Clinical illness scoring system used for this study.

**Score**	**Description**
0 = Healthy	Normal, healthy behavior
1 = Mild	May stand isolated with its head down or ears drooping; but, will quickly respond to minimal stimulation
2 = Moderate	May remain recumbent or stand isolated with head down; may show signs of muscle weakness (standing cross-legged, knuckling, or swaying when walking), depression obvious when stimulated
3 = Severe	May be recumbent and reluctant to rise, or if standing isolated and reluctant to move; when moving, is ataxic, knuckling or swaying evident; head carried low with ears drooping; eyes dull, excess salivation/lacrimation possible, obviously gaunt
4 = Moribund	Unable to stand; approaching death; highly unlikely to respond to any antimicrobial treatment

### Enrollment Procedure

A steer with CIS of 1, 2, or 3 was brought to a processing chute where its rectal temperature was measured. If that temperature met the criterion for the case-definition, a WS was obtained. Two levels of stratification were used based on the WS. Stratum 1 was comprised of steers with WS = 1; stratum 2 was comprised of steers with WS ≥ 2. These stratums were determined based upon the prevalence of each score category observed in field settings (data not shown). Within each stratum, steers were randomly assigned to one of 4 treatment groups in a 2 × 2 factorial design (see Table 2). On a given day (i.e., block), only full treatment blocks, per strata, were enrolled and commingled within pen. Steers that met one of the BRD case definitions but failed to fill all 4 treatments were no longer eligible for enrollment. When a steer met the inclusion criteria and was enrolled, it was weighed and treated as assigned (Table 2). Steers assigned to be negative controls (no treatment administered) were treated with sterile saline (1 mL 0.9% NaCl/cwt, SC). A steer was not eligible for enrollment in the study if it had an unacceptable health condition; had a CIS, a rectal temperature, or a WS outside the criteria stated; or if a full treatment block was not filled.

**Table 2:** Two strata (WS = 1; or WS > 2) were created based on the Whisper Score (WS) at the time of enrollment.

			**Flunixin transdermal solution^b^**	
			**Yes**	**No**
WS = 1	Tildipirosin^a^	Yes	340	340
		No (PSS^c^)	340	170
WS > 2	Tildipirosin^a^	Yes	340	340
		No (PSS^c^)	340	170

*Within the respective stratum, a 2 × 2 factorial (binomial) design was used for assignment to experimental treatment. Two thousand three hundred eighty (2,380) calves were randomly assigned to one of the treatments described below based upon their individual WS at the time of enrollment*.

a *Zuprevo®18% (180 mg tildipirosin/mL) solution; 4 mg tildipirosin/kg (1 mL/cwt) BW, SC*.

b *Banamine Transdermal® (50 mg flunixin meglumine/mL); 3.33 flunixin meglumine/kg (1 mL/15 kg) BW, applied to dorsal midline of back between withers and tailhead*.

c *PSS, Physiologic Saline Solution; 0.9% NaCl; 1 mL/cwt, SC*.

### Post-enrollment Procedures

Daily throughout the study general health of the steers was observed by trained personnel who were blinded to treatments. All adverse events were reported by the same personnel. After enrollment a 2-day post-treatment interval (PTI) was imposed on all treatment groups (including negative controls). Steers were then eligible for additional diagnostic procedures and for retreatment (Table 3). The BRD retreatment case definition reflected a CIS of 1 or 2 and a rectal temperature ≥104° F, or a CIS = 3 regardless of rectal temperature across all treatment groups within both WS strata. Steers with a CIS = 4 were eligible for removal and/or euthanasia at the discretion of the Study Investigator. After the third treatment, a steer was retained in the pen of origin unless its well-being was in jeopardy, it required additional treatment, or it died in the pen. If any of those situations occurred, the steer was removed from the pen, weighed, and removed from the study. If a steer was found dead or was euthanized, a necropsy was performed by authorized personnel. Data pertaining to the individual, to the day of removal, were retained for analysis. Values for the response variables were recorded to Day 60. On Day 61, steers enrolled in the study were moved to larger pens where they were commingled with calves that were not in the study. At closeout (average: Day 274; range: Day 255–281) all steers enrolled in the study were transported to one of three facilities where they were commercially harvested and processed. No final body weights were captured; however, an “adjusted final live weight” was estimated using a carcass yield of 63%.

**Table 3:** Protocol for 2nd and 3rd treatment of BRD if needed.

**BRD event**	**Antimicrobial**	**Dose (mL/cwt)**	**PTI (days)**
2	Nuflor®	6	3
		[mg/kg]	
3	Baytril® 100	5.5	Considered chronic BRD
		[mg/kg]	

The 1st treatment was that assigned when the steer was enrolled in the study. No more than 3 treatments were allowed.

### Design and Analysis

A stratified, randomized, 2 × 2 factorial design was used. Two (2) strata were determined *a priori* and were based on WS. Stratum 1 was comprised of steers with a WS = 1; and, Stratum 2 was comprised of steers with a WS ≥ 2 (Table 2). Within each stratum, a 2 × 2 factorial design was applied with two (2) levels of “tildipirosin; TIL” and two (2) levels of “Flunixin transdermal solution; FTS” comprising those factorials. Descriptive statistics were generated (SAS, version 9.4; Cary, NC) for CIS, rectal temperature, and incoming body weight. Inferential statistics were generated (SAS, version 9.4; Cary, NC) for the dependent/outcome variables which included the following: BRD retreatment risk, days on feed at BRD retreatment, BRD 3rd treatment risk, BRD case-fatality risk, removal risk, did not finish risk (DNF; a combination of both calves that died or were removed due to BRD), and average daily gain (ADG). Steers were randomly assigned to one of those experimental treatments. Statistical analyses were performed using generalized linear mixed models that were fitted using binomial (proportional outcomes; PROC GLIMMIX), multinomial (ordinal carcass grades; PROC GLIMMIX), or normal (continuous outcomes, PROC MIXED) distributions. Degrees of freedom were adjusted via Kenward-Roger estimation. A random intercept was included in all models to account for potential clustering effects within the design structure (lack of independence between the 2 study-sites, among pens within each site, and treatment groups within each pen). Random effects for all carcass metrics included an effect for the plant in which harvest occurred. Treatment group was included as the fixed effect. Enrollment body weight and rectal temperatures were evaluated for differences across treatments between both strata. If associations were observed, the respective independent variable was included in the model statement as a covariate.

## Results

No adverse events associated with the products used were observed during this study.

**Figure 1:**
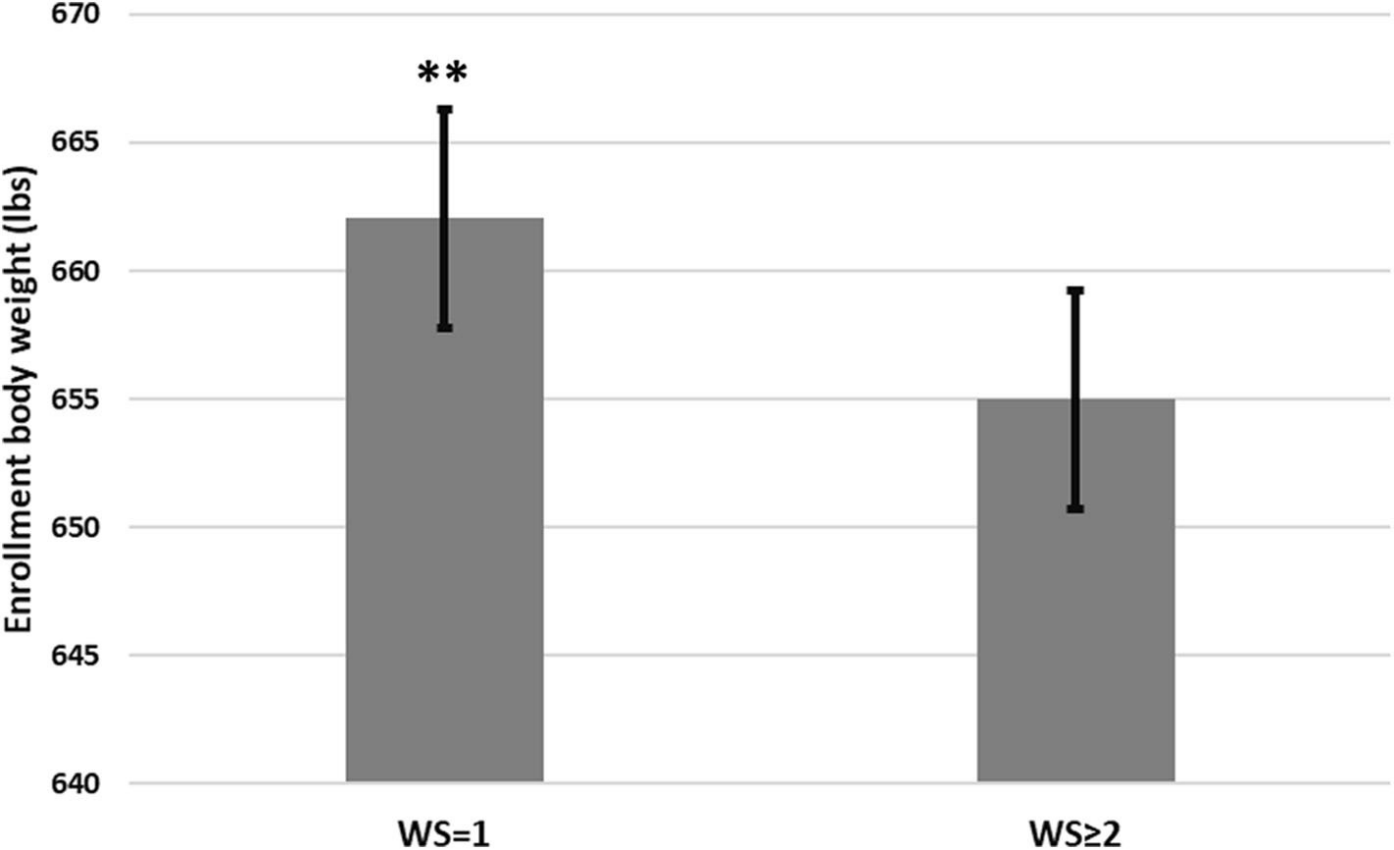
Body weight at enrollment between mixed-breed beef feedlot steers meeting the case definition* of bovine respiratory disease (BRD) with a Whisper Score (WS) of 1 compared to steers with a WS ≥ 2. Error bars denote 95% confidence intervals. *The BRD case definition consisted of an animal displaying a clinical illness score of 1–3 and a rectal temperature of >102.5° F and <103.9° F. A WS was subsequently captured and calves were stratified by WS = 1 or >2. **A statistical difference of *P* < 0.05.

**Figure 2:**
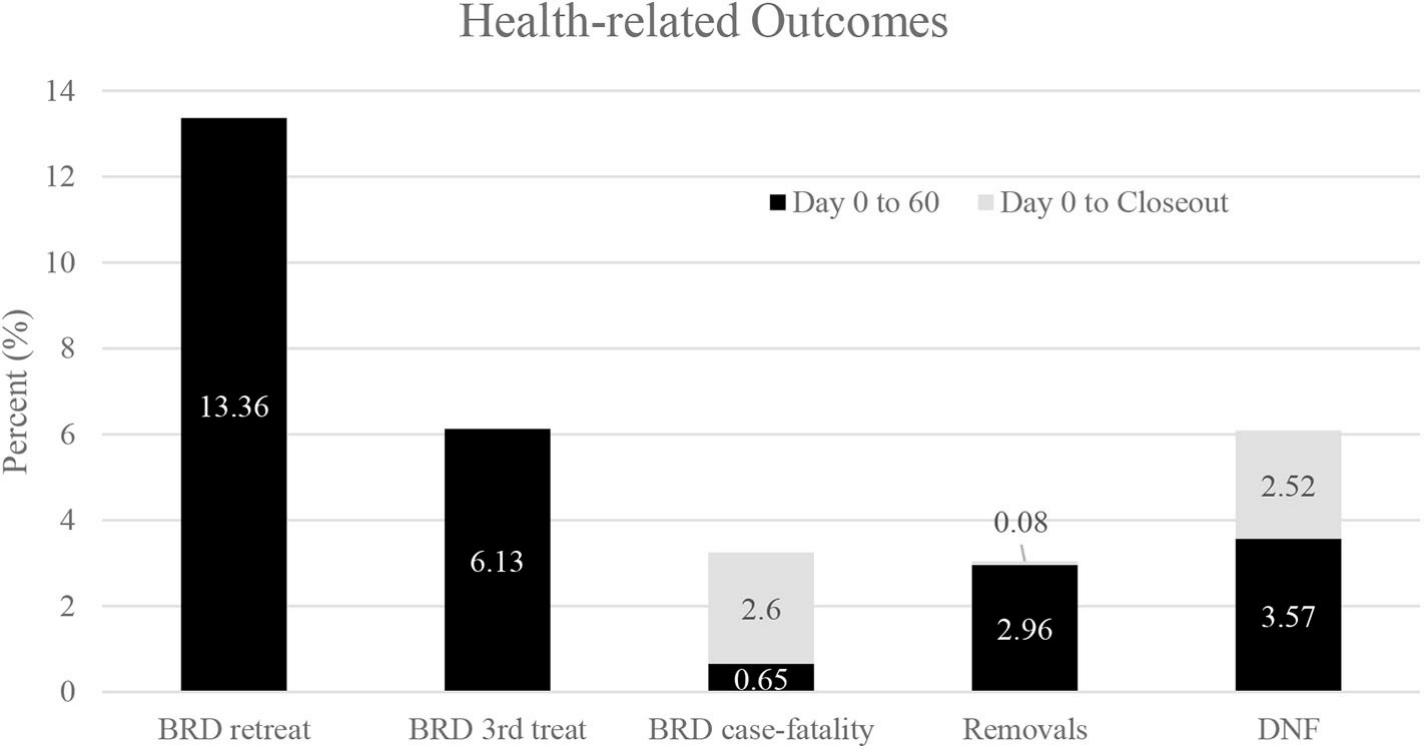
Descriptive statistics for health-related outcomes for all steers from Day 0 to Day 60; and, from Day 0 to closeout. Values for BRD retreatments or for 3rd treatments were not retained after Day 60.

Three thousand, three hundred seventy-six (3,376) steers comprised the pool from which 2,380 steers (70.5% of the pool) were enrolled in this study at two sites (NE = 1,708 steers; KS = 672 steers). Descriptive statistics for those steers are presented in Table 4. Steers with a WS = 1 at the time of enrollment were heavier (*P* < 0.05) compared to steers possessing a WS ≥ 2 (Figure 1). Additionally, although minimal, steers with a WS = 1 at the time of enrollment displayed a reduced rectal temperature (*P* < 0.05; 103.03, 95% confidence interval [95% CI]; 103.01, 103.05) compared to steers possessing a WS ≥ 2 (103.07, 95% CI; 103.05, 103.09). Descriptive statistics for health-related outcomes from Day 0 to Day 60; and, from Day 0 to closeout are presented in Figure 2.

**Table 4:** Descriptive statistics of steers at time of enrollment.

Pool (hd)	3,376
Enrolled by site (hd)	NE = 1,708 KS = 672
Enrolled total (hd)	2,380
CIS 1 (hd)	2,149
CIS 2 (hd)	230
CIS 3 (hd)	1
Average body weight (lb) [range]	658.1 lb [418–938]
Average rectal temperature (^°^F) [range]	103.0 ^°^F [102.5–103.9]

**Figure 3:**
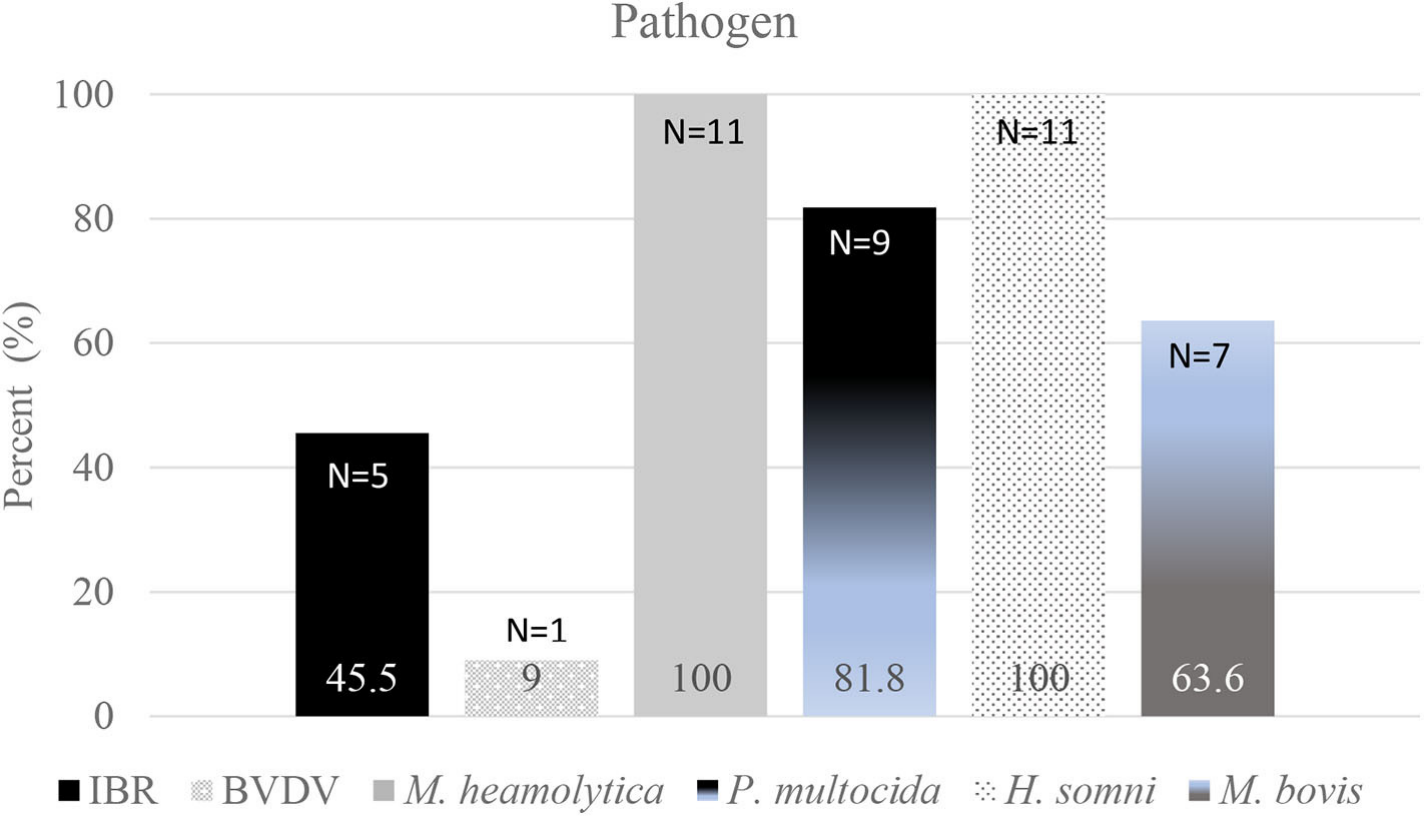
Results of rT-PCR tests to identify pathogens in samples of lung from 11 of 15 steers that died during Day 0 to Day 60. Parainfluenza Type 3 (PI3), BRSV, or BIV were not identified in any sample.

Fifteen (15) steers died due to BRD during Day 0 to Day 60. Samples of lungs from 11 of those 15 (73.3%) were submitted for detection of pathogens using rT-PCR. Of those 11 steers, nine were housed in one pen, and two were housed in one other pen. Results of rT-PCR tests to identify pathogens in samples of lung from those steers are shown in Figure 3. Parainfluenza Type 3 (PI3), BRSV, or BIV were not identified in any sample. Non-BRD conditions resulting in deaths or euthanasia were dietary acidosis (*N* = 1), fibrinous peritonitis (hardware; *N* = 1), severe lameness (*N* = 2), and injury (*N* = 1). Between Day 0 and Day 60 the average day of death due to BRD was Day 29; the average day of death from Day 0 to closeout was Day 135.

Model-adjusted estimates for health-related outcomes and for ADG at the end of the study on Day 60 are presented in Tables 5A,B. Within each stratum (WS = 1 vs. WS ≥ 2), tildipirosin resulted in fewer (*P* ≤ 0.05) BRD retreatments, longer interval to BRD retreatment, fewer BRD 3rd treatments, fewer BRD removals (steers removed but did not die), fewer steers that did not finish (steers that died or were removed), and greater ADG than did steers that were not treated with tildipirosin.

**Table 5A:** Model-adjusted means^*^ (SEM) and corresponding *P*-values for health-related outcomes and ADG from Day 0 to Day 60 by treatment group for steers with a WS = 1.

**Parameter**	**Saline^**a**^**	**FTS^**a**^**	**TIL^**a**^**	**TIL + FTS^**a**^**	***P*-value FTS^**α**^**	***P*-value TIL^**β**^**	***P*-value TIL + FTS^**δ**^**
Incoming body weight (lbs)	663.7 (5.5)	660.0 (4.0)	663.4 (4.0)	660.8 (4.0)	0.48	0.95	0.90
BRD retreatment (%)	15.0 (2.8)	18.5 (2.2)	10.4 (1.7)	8.2 (1.5)	0.95	<0.01	0.17
Day on Feed (DOF) at BRD retreatment (%)	21.6 (3.6)	23.1 (2.7)	31.6 (3.3)	34.1 (3.3)	0.32	<0.01	0.80
BRD 3rd treatment (%)	7.4 (2.1)	9.9 (1.7)	3.4 (1.0)	3.2 (1.0)	0.68	<0.01	0.48
BRD case-fatality (%)	0.57 (0.6)	1.2 (0.6)	0.49 (0.4)	0.85 (0.5)	0.39	0.76	0.91
Removals (%)	2.4 (1.2)	5.0 (1.2)	1.8 (0.7)	1.2 (0.5)	0.67	0.04	0.17
DNF (%)^b^	2.9 (1.3)	5.8 (1.3)	2.3 (0.8)	2.0 (0.8)	0.41	0.07	0.26
ADG (lbs/day)^c^	3.2 (0.1)	3.0 (0.1)	3.5 (0.1)	3.6 (0.1)	0.59	<0.01	0.06

* *Mixed models with a random effect to account for lack of independence among treatment groups within pens, and pens within 2 different sites. Mean and SEM listed above reflect the interactive means of the model*.

a *Saline, negative control; FTS, Flunixin transdermal solution; TIL, tildipirosin; and TIL + FTS, concurrent administration of TIL and FTS*.

b *DNF, did not finish; a combination of both calves that died or were removed due to BRD from Day 0 to 60 of this study*.

c *ADG, Average Daily Gain*.

** *P-values reflect the overall effect of FTS, TIL, and their interaction, respectively. P-values for each main effect reflect a model-adjusted average between treatments incorporating the product compared to those that do not. For example, the P-value for the FTS main effect reflects the comparison between treatments that implement FTS (i.e., FTS and TIL + FTS) vs. those that do not (i.e., Saline + TIL). Only when the P-value for the interaction is significant (P ≤ 0.05) are direct comparison made between the four treatment groups*.

α *P-value for main effect of Flunixin transdermal solution*.

β *P-value for main effect of tildipirosin*.

δ *P-value for interaction of tildipirosin and Flunixin transdermal solution*.

**Table 5B:** Model-adjusted means^*^ (SEM) and corresponding *P*-values for health-related outcomes and ADG from Day 0 to Day 60 by treatment group for steers with a WS ≥ 2.

**Parameter**	**Saline^**a**^**	**FTS^**a**^**	**TIL^**a**^**	**TIL + FTS^**a**^**	***P*-value FTS^**α**^**	***P*-value TIL^**β**^**	***P*-value TIL + FTS^**δ**^**
Incoming body weight (lbs)	649.5 (5.8)	654.4 (4.2)	653.5 (4.2)	660.0 (4.2)	0.21	0.30	0.86
BRD retreatment (%)	15.9 (3.0)	18.0 (2.2)	10.2 (1.7)	10.0 (1.7)	0.74	<0.01	0.66
Day on Feed (DOF) at BRD retreatment (%)	20.5 (3.5)	20.4 (3.0)	25.1 (3.2)	31.3 (3.1)	0.28	<0.01	0.25
BRD 3rd treatment (%)	6.6 (2.0)	9.8 (1.7)	3.7 (1.0)	3.9 (1.1)	0.39	<0.01	0.48
**BRD case-fatality (%)**	**Model did not converge**						
Removals (%)	4.0 (1.5)	5.8 (1.3)	1.8 (0.7)	1.8 (0.7)	0.63	<0.01	0.60
DNF (%)^b^	5.2 (1.7)	6.1 (1.3)	2.3 (0.8)	1.8 (0.7)	0.86	<0.01	0.50
ADG (lbs/day)^c^	3.2 (0.1)	3.0 (0.1)	3.5 (0.1)	3.6 (0.1)	0.58	<0.01	0.10

* *Mixed models with a random effect to account for lack of independence among treatment groups within pens, and pens within 2 different sites*.

a *Saline, negative control; FTS, Flunixin transdermal solution; TIL, tildipirosin; and TIL + FTS, concurrent administration of TIL and FTS*.

b *DNF, did not finish; a combination of both calves that died or were removed due to BRD from Day 0 to 60 of this study*.

c *ADG, Average Daily Gain*.

** *P-values reflect the overall effect of FTS, TIL, and their interaction, respectively. *P*-values for each main effect reflect a model-adjusted average between treatments incorporating the product compared to those that do not. For example, the *P*-value for the FTS main effect reflects the comparison between treatments that implement FTS (i.e., FTS and TIL + FTS) vs. those that do not (i.e., Saline + TIL). Only when the *P*-value for the interaction is significant (*P* ≤ 0.05) are direct comparison made between the four treatment groups*.

*Model did not converge: Lack of enough observations from Day 0 to 60 to generate a model-adjusted estimate*.

α *P-value for main effect of Flunixin transdermal solution*.

β *P-value for main effect of tildipirosin*.

δ *P-value for interaction of tildipirosin and Flunixin transdermal solution*.

Model-adjusted estimates for BRD case-fatality, removals, steers that did not finish, and adjusted-ADG from Day 0 to closeout were analyzed and results are presented in Tables 6A,B. Steers with a WS ≥ 2 that were treated with tildipirosin had fewer removals (*P* < 0.05), and fewer steers that did not finish (*P* < 0.05) compared to those that were not treated with tildipirosin. That was not observed for steers in stratum WS = 1. For steers in WS = 1, there was an interaction (tildipirosin x FTS; *P* ≤ 0.05) for steers treated concurrently with tildipirosin and FTS; and, fewer removals (*P* ≤ 0.05) and fewer steers that did not finish (*P* ≤ 0.05) than did steers treated with only FTS.

**Table 6A:** Model-adjusted means^*^ (SEM) and corresponding *P*-values for health-related outcomes and ADG from Day 0 to closeout by treatment group for steers with a WS = 1.

**Parameter**	**Saline^**1**^**	**FTS^**1**^**	**TIL^**1**^**	**TIL + FTS^**1**^**	***P*-value FTS^**α**^**	***P*-value TIL^**β**^**	***P*-value TIL + FTS^**δ**^**
BRD case-fatality (%)	3.5 (1.4)	4.2 (1.1)	4.0 (1.1)	2.6 (0.9)	0.73	0.64	0.35
Removals (%)	2.4 (1.2)^a^	5.2 (1.2)^a, b^	3.7 (1.0)^a^	1.2 (0.6)^a, c^	0.66	0.19	0.02**
DNF (%)^2^	5.7 (1.8)^a^	8.7 (1.6)^a, b^	7.4 (1.5) ^a^	3.8 (1.0)^a, c^	0.61	0.25	0.03
Adjusted-ADG (lbs/day)^3^	3.3 (0.04)	3.3 (0.03)	3.3 (0.03)	3.3 (0.03)	0.39	0.36	0.51

* *Mixed models with a random effect to account for lack of independence among treatment groups within pens, and pens within 2 different sites*.

** *Different superscripts denote significant differences (P ≤ 0.05) between treatment groups; Pairwise comparisons were only evaluated if the interaction effect (TIL + FTS) was observed to be statistically significant (P ≤ 0.05). All pairwise comparisons were adjusted for multiple comparisons (Tukey method)*.

1 *Saline, negative control; FTS, Flunixin transdermal solution; TIL, tildipirosin; and TIL + FTS, concurrent administration of TIL and FTS*.

2 *DNF, did not finish; a combination of both calves that died or were removed due to BRD from Day 0-closeout of this study*.

3 *Adjusted-ADG: adjusted Average Daily Gain based on a 63% carcass yield*.

** *P-values reflect the overall effect of FTS, TIL, and their interaction, respectively. P-values for each main effect reflect a model-adjusted average between treatments incorporating the product compared to those that do not. For example, the P-value for the FTS main effect reflects the comparison between treatments that implement FTS (i.e., FTS and TIL+FTS) vs. those that do not (i.e., Saline + TIL). Only when the P-value for the interaction is significant (P ≤ 0.05) are direct comparison made between the four treatment groups*.

α *P-value for main effect of Flunixin transdermal solution*.

β *P-value for main effect of tildipirosin*.

δ *P-value for interaction of tildipirosin and Flunixin transdermal solution*.

**Table 6B:** Model-adjusted means^*^ (SEM) and corresponding *P*-values for health-related outcomes and ADG from Day 0 to closeout by treatment group for steers with a WS > 2.

**Parameter**	**Saline^**a**^**	**FTS^**a**^**	**TIL^**a**^**	**TIL + FTS^**a**^**	***P*-value FTS^**α**^**	***P*-value TIL^**β**^**	***P*-value TIL + FTS^**δ**^**
BRD case-fatality (%)	3.1 (1.4)	3.4 (1.0)	3.3 (1.0)	1.5 (0.7)	0.54	0.50	0.44
Removals (%)	5.3 (1.8)	6.3 (1.4)	1.8 (0.7)	2.1 (0.8)	0.66	<0.01	0.95
DNF (%)^b^	8.8 (2.1)	9.4 (1.6)	5.0 (1.2)	3.5 (1.0)	0.68	<0.01	0.31
Adjusted-ADG (lbs/day)^c^	3.3 (0.05)	3.3 (0.03)	3.3 (0.03)	3.3 (0.03)	0.94	0.11	0.79

* *Mixed models with a random effect to account for lack of independence among treatment groups within pens, and pens within 2 different sites*.

a *Saline, negative control; FTS, Flunixin transdermal solution; TIL, tildipirosin; and TIL + FTS, concurrent administration of TIL and FTS*.

b *DNF, did not finish; a combination of both calves that died or were removed due to BRD from Day 0-closeout of this study*.

c *Adjusted-ADG: adjusted Average Daily Gain based on a 63% carcass yield*.

** *P-values reflect the overall effect of FTS, TIL, and their interaction, respectively. *P*-values for each main effect reflect a model-adjusted average between treatments incorporating the product compared to those that do not. For example, the *P*-value for the FTS main effect reflects the comparison between treatments that implement FTS (i.e., FTS and TIL+FTS) vs. those that do not (i.e., Saline + TIL). Only when the P-value for the interaction is significant (*P* ≤ 0.05) are direct comparison made between the four treatment groups*.

α *P-value for main effect of Flunixin transdermal solution*.

β *P-value for main effect of tildipirosin*.

δ *P-value for interaction of tildipirosin and Flunixin transdermal solution*.

Results of analyses of carcass characteristics are presented in Tables 7A,B. For yield and quality grades, data were available for approximately 75% of the steers across all treatment groups within each stratum (data not shown). In stratum WS = 1, tildipirosin resulted in higher marbling scores (*P* ≤ 0.05) and thicker backfat (*P* = 0.08) than did steers not treated with tildipirosin. In stratum WS ≥ 2, steers treated with tildipirosin displayed higher (*P* = 0.08) hot carcass weight (HCW) than did steers that were not treated with tildipirosin.

**Table 7A:** Model-adjusted^*^ means (SEM) and corresponding *P*-values for carcass characteristics by treatment group for steers with a WS = 1.

**Parameter**		**Saline^**1**^**	**FTS^**1**^**	**TIL^**1**^**	**TIL + FTS^**1**^**	***P*-value FTS^**α**^**	***P*-value TIL^**β**^**	***P*-value TIL + FTS^**δ**^**
HCW^2^, lbs		924.4 (6.8)	915.2 (4.9)	926.2 (4.9)	922.7 (4.9)	0.25	0.39	0.61
Ribeye area		15.1 (0.2)	15.3 (0.2)	15.3 (0.2)	15.3 (0.1)	0.45	0.58	0.86
Marbling		462.8 (11.1)	458.2 (8.0)	487.6 (8.0)	482.8 (7.8)	0.59	<0.01	0.99
Backfat		0.57 (0.02)	0.55 (0.01)	0.58 (0.01)	0.60 (0.01)	0.68	0.08	0.26
Calculated Yield Grade		2.9 (0.1)	2.6 (0.1)	3.0 (0.1)	2.9 (0.1)	0.09	0.12	0.29
^a^Yield Grade (*N* = 891)***	1	5.3% (7)	9.9% (25)	10.7% (27)	7.5% (19)	**Model did not converge		
	2	39.8% (53)	39.5% (100)	42.1% (106)	39.5% (100)			
	3	38.3% (51)	36.8% (93)	36.1% (91)	39.9% (101)			
	4	12.8% (17)	12.6% (32)	10.7% (27)	11.5% (29)			
	5	3.8% (5)	1.2% (3)	0.4% (1)	1.6% (4)			
	*N*	133	253	252	253			
^a^Quality Grade (*N* = 894)	Prime	2.3% (3)	2.4% (6)	1.6% (4)	2.7% (7)	0.73	0.81	0.66
	Choice	61.8% (81)	65.0% (165)	62.9% (158)	59.3% (153)			
	Select	32.8% (43)	30.3% (77)	31.5% (79)	32.2% (83)			
	Other	3.1% (4)	2.4% (6)	4.0% (10)	5.8% (15)			
	*N*	131	254	251	258			

* *Mixed models with a random effect to account for lack of independence among treatment groups within pens, pens within 2 different sites, and sites within 3 different processing plants*.

a *Each cell within the Yield and Quality grade outcomes reflects the raw proportions and counts for each treatment group*.

** *Insufficient observations to generate a model-adjusted estimate within the hierarchical structure of the model. Due to non-convergence of the model, the effect of “processing plant” was removed from the random effect and was included as a covariate in the model for “yield grade”. However, the model still did not converge within this WS stratum*.

*** *Total number of steers in each treatment group for Yield and Quality Grade is specified. The proportion of missing data was the same for all treatment groups*.

1 *Saline, negative control; FTS, Flunixin transdermal solution; TIL, tildipirosin; and TIL + FTS, concurrent administration of TIL and FTS*.

2 *HCW, Hot Carcass Weight*.

** *P-values reflect the overall effect of FTS, TIL, and their interaction, respectively. P-values for each main effect reflect a model-adjusted average between treatments incorporating the product compared to those that do not. For example, the P-value for the FTS main effect reflects the comparison between treatments that implement FTS (i.e., FTS and TIL + FTS) vs. those that do not (i.e., Saline + TIL). Only when the P-value for the interaction is significant (P ≤ 0.05) are direct comparison made between the four treatment groups*.

α *P-value for main effect of Flunixin transdermal solution*.

β *P-value for main effect of tildipirosin*.

δ *P-value for interaction of tildipirosin and Flunixin transdermal solution*.

**Table 7B:** Model-adjusted^*^ means (SEM) and corresponding *P*-values for carcass characteristics by treatment group for steers with a WS ≥ 2.

**Parameter**		**Saline^**1**^**	**FTS^**1**^**	**TIL^**1**^**	**TIL + FTS^**1**^**	***P*-value FTS^**α**^**	***P*-value TIL^**β**^**	***P*-value TIL + FTS^**δ**^**
HCW^2^, lbs		912.6 (7.2)	914.5 (5.1)	920.4 (5.0)	926.5 (5.0)	0.48	0.08	0.72
Ribeye area		15.2 (0.2)	15.3 (0.1)	15.3 (0.1)	15.4 (0.1)	0.52	0.51	0.94
Marbling		475.7 (10.3)	481.6 (7.4)	470.2 (7.2)	472.3 (7.1)	0.62	0.36	0.82
Backfat		0.57 (0.02)	0.56 (0.01)	0.56 (0.01)	0.59 (0.01)	0.57	0.60	0.40
Calculated Yield Grade		2.9 (0.1)	2.9 (0.1)	2.8 (0.1)	3.0 (0.1)	0.44	0.87	0.26
^a^Yield Grade (*N* = 902)	1	9.4% (12)	9.7% (25)	11.8% (30)	10.3% (27)	0.69	0.83	0.42
	2	32.0% (41)	38.5% (99)	38.0% (97)	34.4% (90)			
	3	49.2% (63)	38.5% (99)	40.8% (104)	43.1% (113)			
	4	8.6% (11)	11.3% (29)	9.0% (23)	9.9% (26)			
	5	0.8% (1)	1.9% (5)	0.4% (1)	2.3% (6)			
	N	128	257	255	262			
^a^Quality** Grade (*N* = 902)	Prime	1.6% (2)	5.8% (15)	1.2% (3)	3.5% (9)	0.19	0.70	0.32
	Choice	64.1% (82)	62.0% (160)	67.2% (172)	65.4% (170)			
	Select	32.8% (42)	29.1% (75)	27.7% (71)	27.3% (71)			
	Other	1.6% (2)	3.1% (8)	3.9% (10)	3.8% (10)			
	*N*	128	258	256	260			

* *Mixed models with a random effect to account for lack of independence among treatment groups within pens, pens within 2 different sites, and sites within 3 different processing plants*.

a *Each cell within the Yield and Quality grade outcomes reflects the raw proportions and counts for each treatment group*.

** *Total number of steers in each treatment group for Yield and Quality Grade is specified. The proportion of missing data was the same for all treatment groups*.

1 *Saline, negative control; FTS, Flunixin transdermal solution; TIL, tildipirosin; and TIL + FTS, concurrent administration of TIL and FTS*.

2 *HCW, Hot Carcass Weight*.

** *P-values reflect the overall effect of FTS, TIL, and their interaction, respectively. P-values for each main effect reflect a model-adjusted average between treatments incorporating the product compared to those that do not. For example, the P-value for the FTS main effect reflects the comparison between treatments that implement FTS (i.e., FTS and TIL + FTS) vs. those that do not (i.e., Saline + TIL). Only when the P-value for the interaction is significant (P ≤ 0.05) are direct comparison made between the four treatment groups*.

α *P-value for main effect of Flunixin transdermal solution*.

β *P-value for main effect of tildipirosin*.

δ *P-value for interaction of tildipirosin and Flunixin transdermal solution*.

## Discussion

A traditional diagnosis of BRD is based on the presence of clinical signs of the disease, and a rectal temperature ≥104° F, before treatment is prescribed. Results of this study indicate that steers with clinical signs of BRD and rectal temperature <104° F also respond favorably to antimicrobial treatment. In this study tildipirosin resulted in beneficial outcomes that were measurable from Day 0 to Day 60, and from Day 0 to closeout. There were no statistical benefits (or detriments) for steers treated with FTS alone or concurrently with tildipirosin. In stratum WS = 1, a greater proportion of steers treated with FTS alone did not finish compared to steers treated with FTS + tildipirosin. However, no differences were observed among FTS, Saline, and Tildipirosin across these specific outcomes. Additionally, the use of FTS, alone or in conjunction with tildipirosin, did not augment the outcome among calves in either WS strata.

The normal body temperature of beef cattle ranges from 98 to 102.4° F (20). Therefore, given the diurnal variation in body temperature and the cross-sectional nature of rectal temperature data collection, it is likely that a subpopulation of calves presumptively identified with clinical signs of BRD will possess a rectal temperature <104° F. Response to therapy across both Whisper strata among calves with a rectal temperature <104° F may reflect the reality that current BRD diagnostic modalities involve a one point in time event and do not robustly describe the clinical severity of the individual animal. Theurer et al. observed no direct relationship between rectal temperature at the time of the first BRD treatment and the animal's probability of finishing the feedlot phase of production (21). Rather, this relationship was influenced by additional parameters including time of year, gender, and the days on feed prior to first BRD diagnosis (21). These variables likely contribute to the poor diagnostic performance of current BRD diagnostic methods (12, 13).

In this study, the Whisper technology was used in conjunction with traditional diagnostic modalities (clinical signs and rectal temperature) to delineate severity of BRD and to potentially aid in the BRD treatment decision. Since a WS cannot be randomly assigned to an animal, inferences cannot be made between treatments across WS strata; conversely, those decisions can only be made within the chosen strata analyzed in the present study. Although prior studies have shown an association between a rising Whisper Score and worsening lung health (18, 19) and calves enrolled with a WS ≥ 2 were lighter compared to calves with a WS = 1, the data generated in this study indicates that regardless of lung health status, calves in both WS strata require antimicrobial therapy to significantly reduce the risk of BRD relapse. This raises the question as to how to leverage the WS data. As an observation, the positive effects of tildipirosin observed at the 60-day mark within the WS = 1 stratum were not observed at closeout (Table 6A). Conversely, a significant tildipirosin effect was observed in the WS ≥ 2 strata for both the removal and DNF outcomes from Day 0 to closeout (Table 6B). This may suggest that calves identified with clinical signs of BRD, a rectal temperature <104° F and a WS = 1 are more likely to finish the feeding phase of production compared to calves with a WS ≥ 2 and a rectal temperature <104° F at the time of first BRD diagnosis. This observation among calves with a WS = 1 raises two potential opportunities to utilize these data: (1) Given that calves in both WS strata responded positively to antimicrobial therapy, perhaps a less potent (and cheaper) antimicrobial may be applicable in this subpopulation of cattle stricken with BRD; and (2) perhaps the value of the WS may be realized in the subsequent management of calves with more severe lung health issues at the time of first BRD diagnosis rather than impacting antimicrobial treatment decisions. More work is necessary to test these theories.

One potential limitation of this study is that the study population was not maintained within their original cohort from the time of enrollment until closeout. Rather, they were placed in a larger general population after 60 days post-BRD diagnosis. This occurrence may have led to an underestimation of overall BRD morbidity due to further reduction in BRD diagnostic accuracy within a larger group size. Additionally, final body weights were not captured to avoid unnecessary stress on the finished animals which necessitated back calculation and estimation of final weights based on HCW and carcass yields (63%). This likely had an impact on the precision of the ADG estimates at closeout. However, these management decisions were applied universally across the study population and individual identification was accurately maintained through closeout.

In this study, calves exhibiting clinical signs of BRD but with rectal temperatures <104° F (regardless of WS) responded favorably to tildipirosin therapy at the time of first BRD diagnosis. Withholding antimicrobial medication from feedlot animals that have not reached the traditional threshold for rectal temperature may not be prudent for the best interest of the animal or the producer.

## Data Availability Statement

The datasets presented in this article are not readily available because the study data are owned by Merck Animal Health. Access to these data would require additional approval beyond that of the authors. Requests to access the datasets should be directed to Dr. John Hutcheson, john.hutcheson@merck.com.

## Ethics Statement

This animal study was reviewed and approved by the study protocol review committee consisted of an independent group compiled by authors CC and KL.

## Author Contributions

This study was designed and the protocol generated by JN and LB. This study was executed by CC and KL and monitored by JN and LB. Statistical analysis was performed by JN. Manuscript writing reflected a collaboration amongst all authors.

## Conflict of Interest

This study was fully funded by Merck Animal Health (MAH). Allflex Livestock Intelligence (ALI) is a subsidiary of MAH. JN and LB (employees of ALI and MAH, respectively) were responsible for study design, monitoring, and data analysis. Midwest Veterinary Services (MVS) and Veterinary Biomedical Research Center (VBRC) are contract research organizations wholly owned by KL that provides professional services to support the regulatory research required by FDA, USDA, and EPA for approval of animal health products. MVS's Institution Animal Care and Use Committee (IACUC) is responsible for oversight of the animal program and its components as described in the USDA and PHS guidelines. MVS continues to pass annual to bi-annual audits from government regulatory agencies. The study was executed under the oversight of KL and CC. The study protocol was submitted to the MVS IACUC where the protocol received approval on 30 January 2018. The assigned IACUC number is AC17100B.
